# Long-term neurocognitive outcome is not worsened by of the use of venovenous ECMO in severe ARDS patients

**DOI:** 10.1186/s13613-019-0556-1

**Published:** 2019-07-16

**Authors:** Aude Sylvestre, Mélanie Adda, François Maltese, Ariane Lannelongue, Florence Daviet, Gabriel Parzy, Benjamin Coiffard, Antoine Roch, Anderson Loundou, Karine Baumstarck, Laurent Papazian

**Affiliations:** 1 Assistance Publique - Hôpitaux de Marseille, Hôpital Nord, Médecine Intensive Réanimation, Chemin des Bourrely, 13015 Marseille, France; 2 Centre d’Etudes et de Recherches sur les Services de Santé et qualité de vie EA 3279, Faculté de médecine, Aix-Marseille Université, 13005 Marseille, France; 3 Assistance Publique - Hôpitaux de Marseille, Hôpital Nord, Service des Urgences, 13015 Marseille, France; 4 Département d’Anesthésie Réanimation, Centre Hospitalo Universitaire Caremeau, 30029 Nîmes, France; 5 Unité d’Aide Méthodologique à la Recherche Clinique - Assistance Publique - Hôpitaux de Marseille, Marseille, France; 6 EA 3279, Faculté de médecine, Aix-Marseille Université, 13005 Marseille, France

**Keywords:** Cognitive impairment, Long-term neuropsychological outcome, Long-term cognitive and psychiatric morbidity, WAIS-IV, PTSD

## Abstract

**Background:**

Venovenous extracorporeal membrane oxygenation (VV-ECMO) is associated with a significant morbidity. There is the need to investigate long-term cognitive outcome among ARDS survivors treated with VV-ECMO. We aimed to compare the prevalence of long-term cognitive dysfunction and neuropsychological impairment using a highly specific test in severe ARDS survivors treated or not treated with VV-ECMO.

**Methods:**

Severe ARDS survivors treated between 2011 and 2017 in an ECMO Regional Referral Center were prospectively evaluated 2 years after their ICU discharge. Patients underwent an in-person interview and examination. The primary outcome was cognitive function, assessed by the Wechsler Adult Intelligence Scale 4th edition (WAIS-IV). Secondary outcomes included anxiety, depression, post-traumatic stress disorder (PTSD) and quality-of-life.

**Results:**

We investigated 40 consecutive patients surviving from severe ARDS treated (*N* = 22) or not (*N* = 18) with VV-ECMO at a median [interquartile range] of 20 [17–22] and 22 [18–23] months after ICU discharge, respectively. Regarding the main outcome, cognitive function was below normal ranges in 12 (55%) ECMO patients and 10 (56%) non-ECMO patients (*p* = 0.95). Eight (36%) ECMO patients had moderate-to-severe depressive symptoms as compared with 7 (39%) non-ECMO patients (*p* = 0.87). Twelve (55%) ECMO patients and eight (44%) non-ECMO patients had moderate-to-severe anxiety symptoms (*p* = 0.53). Seven (33%) ECMO patients and eight (44%) non-ECMO patients presented a PTSD (*p* = 0.48). Health-related quality of life did not differ between the two groups.

**Conclusions:**

Using the WAIS-IV, VV-ECMO treatment does not appear to worsen long-term cognitive and neuropsychological outcomes in severe ARDS patients.

**Electronic supplementary material:**

The online version of this article (10.1186/s13613-019-0556-1) contains supplementary material, which is available to authorized users.

## Introduction

Venovenous extracorporeal membrane oxygenation (VV-ECMO) is a rescue therapy used in severe ARDS patients to allow lung-protective mechanical ventilation [1] and to provide time for treating the cause of ARDS thus permitting lung healing. It is also a life-saving measure when mechanical ventilation cannot maintain adequate oxygenation or CO_2_ elimination. It remains an unusual treatment (7% of severe ARDS [2]) mainly provided by expert centers [3].

The use of VV-ECMO in patients with severe ARDS has increased dramatically with more than 19,000 cases per year worldwide [4]. Even though its benefits on mortality remains unclear [5–7], hospital survival of patients treated with VV-ECMO in the last 10 years is improving and now averages 59% [4]. Long-term survival as high as 76–87% at 5 years has been reported, especially in patients treated for infection [8, 9].

However, surviving the ICU frequently has a price [10], and ARDS survivors suffer long-lasting significant physical, cognitive [11–14] and psychological sequelae such as PTSD, a depressive mood and anxiety [15–17] with consequences on social functioning, ability to return to work and health-related quality-of-life impairment [18, 19].

Because of its invasiveness, the need for curative anticoagulation, the bleeding risk increased by thrombocytopenia with potential harmful cerebral complications [20, 21]; VV-ECMO might be even more of a risk factor for cognitive dysfunction.

To the best of our knowledge, there is no study comparing the long-term cognitive outcome evaluated by a highly specific test of severe ARDS patients treated with VV-ECMO to a population of severe ARDS patients who did not undergo VV-ECMO. Therefore, we aimed to compare the long-term prevalence and impact of cognitive impairment (and neuropsychological sequelae) in severe ARDS patients treated or not with VV-ECMO. We hypothesized that VV-ECMO does not worsen long-term cognitive outcomes in severe ARDS survivors.

## Methods

### Study design

This cross-sectional comparative cohort study was conducted in the North University Hospital medical ICU, Marseille, France, which is a regional VV-ECMO referral center for acute respiratory failure.

All surviving adult patients treated in the ICU for severe ARDS between May 2011 and March 2017 were prospectively screened for eligibility. Criteria for defining severe ARDS were those of the Berlin definition [22] plus a PaO_2_-to-FiO_2_ ratio (P/F ratio) of less than 100 under mechanical ventilation with a positive end-expiratory pressure (PEEP) of at least 10 cm H_2_O for more than 48 h. Indications for ECMO were a P/F ratio of less than 70 for at least 2 h under a FiO_2_ of 1, a P/F ratio of less than 100 mm Hg associated with a plateau pressure value greater than 35 cm H_2_O or respiratory acidosis with pH less than 7.15 despite a respiratory rate greater than 35/min. Absolute contraindications were any contraindication to heparin treatment, a SOFA score greater than 18 when mechanical ventilation duration was less than 7 days or a SOFA score exceeding 12 when mechanical ventilation was longer than 7 days. Venoarterial ECMO, patients treated with ECMO as bridge to or following lung transplantation, patients with prior psychiatric illnesses or neurocognitive impairments or non-French speakers were not included.

Eligible survivors were contacted by phone, and those who agreed to participate were asked to complete a questionnaire prior to meeting the physician and the psychologist at the hospital or—if impossible—at their home. Patients were evaluated 18–24 months following their ICU discharge after their informed consent was obtained. This study was approved by the Institut Fédératif de Recherche 48 research ethic board of Aix-Marseille university.

### Characteristics of patients and ICU course

All clinical and biological information was prospectively collected during the ICU stay. Day 1 was defined as the first day when severe ARDS criteria were satisfied. (Some patients were in another hospital before being transferred to the referral regional ECMO center.) A full-time psychologist was part of the ICU team and proposed to meet all families and all patients (when recovering). All included patients referred to our center were retransferred while being weaned from ECMO. After sedation has been stopped, these patients were retransferred while they were receiving pressure support mode of ventilation. There was no specific recommendation regarding the post-ICU care, neither for the referring centers nor for the referral center.

### Assessment/follow-up protocol

Patients were evaluated at the Marseille, North University hospital or—if impossible—in their homes. First, they were interviewed by an ICU physician (MA or AS) about their symptoms and activities since hospital discharge. Cognitive function was then assessed by a trained psychologist (FM) using the Wechsler Adult Intelligence Scale, 4th edition (WAIS-IV) [23, 24]. The WAIS-IV test duration was approximately 2 h. With ten main and five additional subtests, the WAIS-IV measures four cognitive domains: verbal comprehension, perceptual reasoning, working memory and processing speed. The verbal comprehension index (VCI) reflects the verbal performance, education and culture level of the patient, independently of attention and focus skills or processing speed. The perceptual reasoning index (PRI) assesses visuo-spatial abilities and cognitive flexibility. The working memory index (WMI) measures the abilities of attention and concentration and problem-solving skills. The processing speed index (PSI) evaluates the performance in speed and accuracy of execution. These 4 indexes are summarized in the Full-Scale Intelligence Quotient (FSIQ) reflecting global cognitive function. The WAIS-IV includes normative data according to gender and ages from 16 to 90.

Patients had to fulfill other validated self-evaluation questionnaires: the shortened Beck Depression Inventory (BDI-IA) for depression [25, 26], the Beck Anxiety Inventory (BAI) [27, 28] for anxiety, the Impact of Event Scale (IES) for PTSD [29, 30] (scores ≥ 36 indicating substantial PTSD symptoms [31]) and the Short-Form General Health Survey (SF-36) for health-related quality of life [32, 33]. The patients could complete the questionnaire by their own at home to shorten the duration of the consultation. However, these questionnaires were carefully reviewed by the psychologist and the physician in the presence of the patients in order to have all the items fulfilled.

### Statistical analysis

Descriptive statistics included percentages for categorical variables and medians and interquartile ranges (IQRs) for continuous variables. According to their distribution, comparisons were made using either the Pearson Chi-square test or the Fisher exact test for categorical variables and the Mann–Whitney *U* test for continuous variables.

According to the intelligence quotient standard scale (mean, 100; SD, 15) [23], impairment was defined as at least one index of the WAIS-IV below one standard deviation (SD). Sensitivity analyses were performed based on other cognitive dysfunction definitions. We performed univariate analyses to evaluate the potential determinants of cognitive dysfunction as previously defined. Significant variables in the univariate analysis (*p* < 0.05) or those clinically relevant were introduced into a logistic regression analysis. The final model provided the odd ratios (OR) and 95% confidence intervals (CI). Statistical analysis was performed using the SPSS statistics 20 software.

## Results

### Patients

**Fig. 1:**
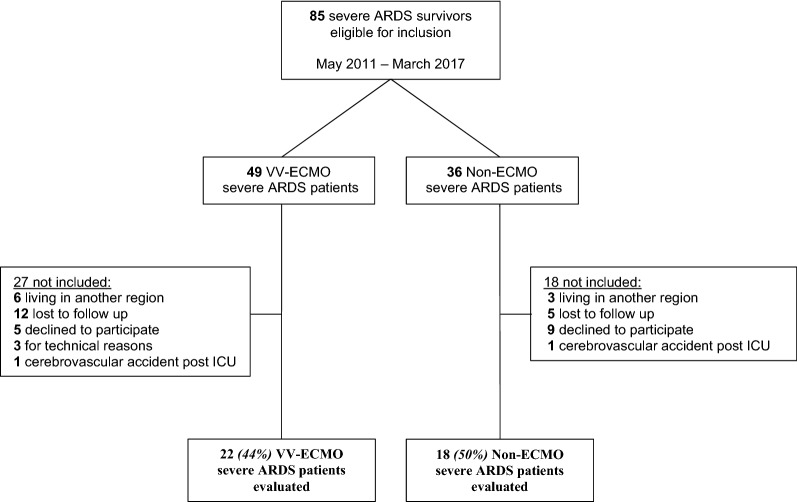
Flowchart

A total of 125 severe ARDS patients were discharged alive from the ICU from May 2011 to March 2017. Four ECMO and seven non-ECMO patients died before the 2-year follow-up. We identified 85 eligible ICU survivors (Fig. 1), 49 of them received VV-ECMO therapy (ECMO group) and 36 did not undergo VV-ECMO (non-ECMO group). Two patients (one in each group) were excluded because of sequelae from a cerebrovascular accident post-ICU discharge. Finally, 45.7% (40 patients) of the eligible patients were included, 22 in the ECMO group and 18 in the non-ECMO group.

Characteristics on ICU admission are presented in Table 1. There was no difference between the two groups except for the education level (patients in the ECMO group had a higher educational level). The cohort was healthy before onset of ARDS as evidenced by high functional status and minimal associated comorbidities. As stated in Table 2, duration of profound hypoxemia with a PaO_2_ lower than 50 mmHg was longer in the ECMO group, as was the duration for PEEP levels higher than 10 cm H_2_O, muscle relaxants and narcotics use. In contrast, there was no difference regarding important outcomes such as the duration of mechanical ventilation, the duration of hospitalization (both in the ICU and in the hospital) and the neuromuscular examination at ICU discharge. More information regarding the evolution of the SOFA score, PaO_2_ and ventilator settings are provided in Additional file 1: Table S1.

**Table 1:** Baseline characteristics of patients at ICU admission

Characteristics	Non-ECMO (*n* = 18)	ECMO (*n* = 22)	*p* Value
Male sex, *n* (%)	11 (61)	12 (55)	0.68
Age, years	51 [43–63]	41 [32–56]	0.89
BMI, kg/m^2^	27 [22–36]	28 [25–31]	0.76
SAPS II score at ICU admission	43 [37–51]	42 [33–53]	0.56
SOFA score at ICU admission	8 [6–12]	8 [7–10]	0.76
McCabe Score at ICU admission ≥ 1, *n* (%)	4 (22)	5 (23)	0.99
College level education, *n* (%)	6 (33)	16 (73)	0.013
Full-time work, *n* (%)	9 (50)	13 (59)	0.57
Coexisting conditions, *n* (%)			
None	2 (11)	3 (14)	0.99
Chronic lung disease	5 (28)	6 (27)	0.99
Chronic cardiac disease	6 (33)	7 (32)	0.92
Vascular disease	1 (6)	3 (14)	0.61
Neurologic disease	0 (0)	2 (9)	0.49
Diabetes mellitus	3 (17)	1 (5)	0.31
Hypothyroidy	1 (6)	2 (9)	0.99
Mild psychiatric disease	2 (11)	1 (5)	0.58
Malignancy	0 (0)	1 (5)	0.99
Immunocompromized	2 (11)	3 (14)	0.99
Smoking habit	7 (39)	9 (41)	0.90
Alcohol abuse	5 (28)	6 (27)	0.99
Drug addiction	1 (6)	2 (9)	0.99
Primary lung injury, *n* (%)			0.33
Bacterial pneumonia	5 (28)	10 (46)	
Influenza pneumonia	3 (17)	7 (32)	
Other viral pneumonia	0 (0)	1 (5)	
Post-operative respiratory failure	4 (22)	3 (14)	
Aspiration pneumonia	1 (6)	0 (0)	
Multiple blood transfusion	1 (6)	0 (0)	
Extrapulmonary sepsis	1 (6)	0 (0)	
Other or unknown	3 (17)	1 (5)	
Transferred from another hospital, *n* (%)	10 (56)	17 (77)	0.15

Data are provided as numbers (%) for categorical variables and as medians [25th–75th percentiles] for continuous variables

*BMI* Body mass index, *ICU* intensive care unit, *ARDS* acute respiratory disease syndrome, *SAPS II* simplified acute physiology score II, *SOFA* sequential organ failure assessment

**Table 2:** Characteristics of the patients during the ICU stay

Characteristic	Non-ECMO (*n* = 18)	ECMO (*n* = 22)	*p* Value
MV duration before ARDS, days	2 [0–5]	0.5 [0–5]	0.66
MV duration before ECMO, days	–	4 [1–9]	
Lung Injury Score at day 1	3 [2.5–3.1]	3 [2.8–3.4]	0.23
Lowest PaO_2_ at day 1, mmHg	69 [62–79]	73 [52–81]	0.93
Lactate level at day 1 (mmol/L)	2.3 [1.4–3.1]	2.2 [1.4–3.1]	0.99
Duration of hypoxemia (PaO_2_ < 50 mmHg), hours	2 [1–5]	6 [3–8]	0.026
Duration of hypoxemia (PaO_2_ < 65 mmHg), hours	10 [6–31]	31 [9–54]	0.09
Days of PEEP > 10 cm H_2_O	13 [8–17]	16 [14–30]	0.022
Adjunctive therapies			
Nitric oxide, *n* (%)	6 (33)	11 (50)	0.29
Prone positioning, *n* (%)	17 (94)	17 (77)	0.20
Days receiving benzodiazepines	14 [11–18]	25 [21–34]	< 0.001
Days receiving narcotics	14 [11–20]	26 [22–35]	< 0.001
Days receiving neuromuscular blockade	7 [3–10]	11 [7–15]	0.022
Days receiving neuroleptics	4 [1–7]	4 [0–11]	0.90
Days receiving vasopressors	7 [3–16]	12 [8–16]	0.08
Delirium during ICU, *n* (%)	12 (67)	12 (55)	0.44
Renal replacement therapy, *n* (%)	5 (27.8)	6 (27.3)	0.99
Transfusion, *n* (%)	16 (89)	22 (100)	0.20
Any systemic glucocorticoid therapy, *n* (%)	12 (67)	17 (77)	0.46
Septic shock, *n* (%)	5 (42)	9 (53)	0.55
ARDS, *n* (%)	1 (8)	6 (35)	0.19
Other, *n* (%)	6 (50)	5 (29)	0.26
No. VAP	0 [0–1]	1 [0–2]	0.6
No. episodes blood glucose < 3.5 mmol/L	0 [0–2]	0 [0–2]	0.93
Natremia < 135 mmol/L, days	3 [1–8]	1 [1–6]	0.33
Natremia > 146 mmol/L, days	4 [2–6]	5 [1–7]	0.78
Cumulative 7-days fluid balance, L	12.5 [7.3–16.4]	12.8 [9.31–22.2]	0.31
MRC neuromuscular score at discharge	42 [33–46]	39 [33–48]	0.70
Duration of ECMO support, days	–	12 [8–19]	
Duration of MV total, days	29 [21–46]	36 [28–64]	0.16
ICU LOS, days	35 [24–47]	46 [34–71]	0.08
Hospital LOS, days	55 [43–90]	61 [45–99]	0.77
Time to return back to home, days	93 [57–123]	120 [72–158]	0.13

Data are provided as numbers (%) for categorical variables and as medians [25th–75th percentiles] for continuous variables

*MV* Mechanical ventilation, *ECMO* extracorporeal membrane oxygenation, *LOS* length of stay, *PEEP* positive end-expiratory pressure, *ICU* intensive care unit, *ARDS* acute respiratory distress syndrome, *VAP* ventilator-acquired pneumonia, *MRC* Medical Research Council

### Cognitive status

The median [interquartile range] follow-up time was 20 [17–22] months post-ICU discharge for the ECMO group and 22 [18–23] months for the non-ECMO group (*p* = 0.35).

Long-term cognitive impairment (defined as at least one WAIS-IV index below one SD) was present in 12 (55%) ECMO and 10 (56%) non-ECMO patients (*p* = 0.95). As shown in Additional file 2: Table S2, there was no difference among the two groups, regardless of the threshold and the indexes used to define cognitive impairment.

**Fig. 2:**
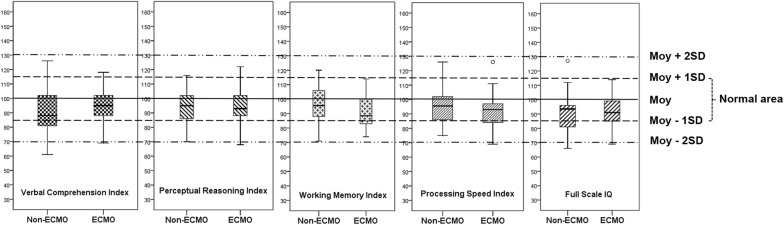
Cognitive function assessed by the Wechsler Adult Intelligence Scale 4th edition (WAIS-IV)

The various indexes assessed by the WAIS-IV are detailed in Fig. 2 and Additional file 3: Table S3. Global cognitive functioning (FSIQ) was within one SD or above the mean of a healthy population in 17 (77%) ECMO patients and 13 (72%) non-ECMO patients. Five ECMO patients (23%) and five non-ECMO patients (28%) had a FSIQ which was one SD or more below the mean (*p* = 0.73). Only one (5%) ECMO patient and one (6%) non-ECMO patient had a FSIQ which two SD or more below the mean (*p* = 0.88).

After adjustment according to the duration of profound hypoxemia, the duration of sedation with benzodiazepines, the cumulative 7-day fluid balance and the duration of hyponatremia (below 135 mmol/L), there was still no significant differences between ECMO and non-ECMO patients regarding cognitive impairment. As the education level was different among the two groups, we also evaluated separately the two WAIS-IV indexes which can be altered by education (i.e., VCI and WMI). This analysis did not show any difference between the ECMO group and the non-ECMO group regarding these two indexes even after adjustment.

### Psychological outcomes

Signs of depression were reported in eight (36%) ECMO patients and seven (39%) non-ECMO patients (*p* = 0.87). Anxiety was identified in 12 (55%) ECMO and eight (44%) non-ECMO patients (*p* = 0.53) (Table 3).

**Table 3:** Neuropsychological tests results

Variables	Non-ECMO (*n* = 18)	ECMO (*n* = 22)	*p* Value
Beck Depression Inventory (BDI-A)	4 [2–12]	5 [2–5, 5–14]	1
Depressive symptoms severity			0.87
Minimal/mild (BDI-A score 0–7)	11 (61)	14 (64)	
Moderate/severe (BDI-A score ≥ 8)	7 (39)	8 (36)	
Beck Anxiety Inventory (BAI)	15 [10–32]	16 [6–29]	0.80
Anxiety symptom severity			0.53
Minimal/mild (BAI score 0–15)	10 (56)	10 (46)	
Moderate/severe (BAI score 16–63)	8 (44)	12 (55)	
Impact of Event Scale (IES)			
Intrusion subscale score	12 [6–17]	17 [5–30]	0.31
Avoidance subscale score	12 [5–21]	4 [2–14]	0.12
Total score	23 [13–36]	18 [9–41]	0.69
Post-traumatic stress disorder (PTSD)	8 (44)	7 (33)	0.48

Data are provided as numbers (%) for categorical variables and as medians [25th–75th percentiles] for continuous variables

A PTSD was present in seven (33%) ECMO patients and in eight (44%) non-ECMO patients (*p* = 0.48) (Table 3).

The most affected quality-of-life domains as assessed by the SF-36 were the physical and emotional role but without between-group differences (Additional file 4: Table S4). A partial correlation was tested between the mental score composite of the SF-36 and the cognitive indices accounting for the group, showing significant associations between MCS and Verbal Comprehension Index (partial correlation coefficient *R* = 0.45, *p* = 0.006), processing speed index (*R* = 0.36, *p* = 0.032) and full scale intelligence index (*R* = 0.40, *p* = 0.016).

### Other outcomes

Additional information including the results of the examination done by the ICU physician is summarized in Additional file 5: Table S5.

Just before hospital admission related to the ARDS, 59% of the ECMO patients (13 of 22) were working full time as compared with 50% of the non-ECMO patients (9 of 18) (*p* = 0.41). At the 2-year follow-up, 46% of these patients from the ECMO group (6 of 13) had returned to their original work as compared with 67% from those included in the non-ECMO group (6 of 9) (*p* = 0.41). Before ARDS, 13 (59%) patients were working full time in the ECMO group versus 9 (50%) in the non-ECMO group. Among those, two (15%) patients have returned to work full time in the ECMO group and two (22%) in the non-ECMO group. Patients who had not returned to work reported being unemployed as a result of their disabilities.

## Discussion

This study assessed cognitive and neuropsychological long-term outcomes in severe ARDS survivors comparing patients treated or not treated with VV-ECMO through the use of an in-person interview using a well-established and validated highly specific measurement instrument. The results of the present study suggest that VV-ECMO treatment does not worsen long-term cognitive and neuropsychological outcomes in severe ARDS survivors.

### External validity

The main concern in comparing neurocognitive outcome studies is the great variability of the measurement instruments used and the lack of validated thresholds to define cognitive impairment. Only two non-comparative ECMO cognitive outcome studies used the WAIS-IV as in the present study. Von Bahr et al. [34] found results similar to our cohort with a median full-scale intelligence index of 97, but the follow-up time was much longer with a mean of 9 years post discharge. Holzgraefe et al. [35] found also similar results at a median of 3.2 years post ICU discharge but with a cohort of only seven patients. However, in these two studies, there was no control group without ECMO.

Moreover, previous similar studies have frequently combined venovenous and venoarterial ECMO [21, 34], whereas the risk of cerebral injury—and hence cognitive outcome—is much greater with venoarterial than venovenous ECMO.

Risnes et al. [21] found an incidence of 33% of cognitive impairment predominant on attention and verbal memory, 5 years after discharge, as well as a correlation between cognitive impairment and neuroradiologic findings. However, they used a composite impairment score in a pediatric and adult population with various indications of ECMO [21].

In the present study, few patients had a cerebral imaging during or after ICU stay; however, this is acceptable since the incidence of cerebrovascular lesion in VV-ECMO patients has declined from 17 to 26% in some reports [21, 34] to 2% in the recent EOLIA trial [7]. Moreover, as there was no difference among the 2 groups, the need for cerebral imaging in highly questionable.

Luyt et al. [36] did the only comparative study and found similar outcomes in terms of health-related quality of life, incidence of anxiety, depression and PTSD in H1N1-ARDS survivors treated with ECMO or conventional ventilation 1 year after their discharge. However, cognitive function was not assessed in this cohort.

Hodgson et al. [37] reported that long-term ARDS survivors treated with ECMO had low general health, mental health, vitality and social function SF-36 subscores, but unlike our study this study was not comparative.

Even though ECMO patients were more subjected to severe hypoxemia in the present report, it was not associated with more cognitive dysfunction, which is consistent with other similar studies [34, 35].

In our cohort, very few patients had returned to work 2 years after their discharge which is inconsistent with other studies. In Luyt’s cohort, 83% of ECMO patients and 64% of non-ECMO patients had returned to work 1 year post discharge from the ICU [36] whereas 53% of patients had returned to work at 8 months in Hodgson’s cohort [37]. This may be explained by the fact that patients were younger in both cohorts [36, 37] as compared with ours.

Cognitive and neuropsychological outcomes of the non-ECMO control group from the present study are consistent with other reports of long-term outcome in ARDS survivors [11, 14, 16, 18, 38]. Health-related quality of life is also comparable to previous studies except for the physical and emotional dimension [12, 14, 39]. It should be noted that we did not compare these results to the SF-36 scores of the healthy French population. However, we believe that these results are consistent with the low proportion of patients who returned to work because physical and emotional aspects reflect the extent to which physical health and emotional problems interfere with work and may limit activity.

### Strengths and limitations

One of the main strengths of the present study is the comprehensive in-person evaluation of cognitive function assessed by an expert. We used validated outcome measurement instruments to assess cognitive and psychological function in ARDS patients. Few ECMO long-term outcomes studies have a control group; the comparative design of this study is another strength.

There are several limitations to this study. The first is the small sample size and the absence of randomization due to the study’s nature as a single-center study. Moderate associations were possibly missed because of low statistical power due to the sample size, which was arguably too small. A larger sample will allow the confirmation of these findings. As the differences were very small, it is highly probable that a larger sample would confirm the present results. The reason why some patients with very profound hypoxemia were treated with VV-ECMO and others were not is related to the admission procedure of the patients. Indeed, our ICU is the regional (a 31,400-km^2^ region with approximately five million inhabitants) VV-ECMO referral center for the treatment of severe respiratory failure. For patients referred to our center, VV-ECMO was implemented in the referring center by our mobile team before transfer. In our own center, as VV-ECMO can be very rapidly applied, we are able to wait until lower PaO_2_ levels. This is confirmed by the information provided in Table 1 showing that 8 of 18 patients from the non-ECMO group were initially hospitalized in our own ICU as compared with only 5 of 22 from the ECMO group. Families play an important role in the outcome of ICU patients, and we did not evaluate between groups differences regarding family support. Moreover, even though psychological symptoms like anxiety and PTSD in patients may affect willingness to participate in such follow-up research and lead to an under estimation of the prevalence of such neuropsychological impairments, follow-up rates in this cohort were rather good with more than 45% of the eligible patients included. Second, although patients with prior neurocognitive or psychological impairment were excluded, we did not have patient’s baseline cognitive data, which is a common limitation of cognitive function ICU studies. We tried to address this by adjusting on the education level. Although ECMO patients had a higher educational level, no significant difference was found between the two groups after multivariate analysis. Also, because working memory and verbal comprehension indexes can be influenced by the level of education, we performed an adjustment on this parameter and found no significant differences between ECMO and non-ECMO groups.

The WAIS-IV is exploring only some aspects of neurocognition. Neurocognitive function also included psychomotor speed, spatial reasoning, verbal memoring, visual memoring which are partially assessed by the WAIS-IV. However, cognitive exploration takes time and is not easy to insert in a prolonged interview. The evaluation proposed in the present study was face-to-face, lasting approximately 3 h, which is already noteworthy in a post-ICU follow-up study. Finally, it is extremely difficult to distinguish normal variations in test performances from true cognitive impairment. In fact, for the WAIS-IV, 68% of the healthy French population scores vary between ± 1 SD of the mean and 95% between ± 2 SD [24]. We performed the statistical analysis with three different thresholds and found no significant differences between the two groups regardless of the cutoff. We finally chose to use a cutoff of at least one WAIS-IV index below one SD to define cognitive impairment even though by doing so we may have overestimated the incidence of cognitive impairment.

### Prevention

This study emphasizes the need to prevent long-term cognitive and neuropsychological sequelae in ARDS patients. ICU-based interventions like ICU diaries written by clinicians and family members to critically ill patients [40], in-ICU psychological and cognitive interventions [41], social support [38], rehabilitation psychologists, post-ICU coping skills training [42] and rehabilitation clinics could be helpful in reducing long-term cognitive and neuropsychological disorders. However, they need a more extensive evaluation.

Follow-up and rehabilitation programs in order to screen, prevent and treat these impairments need to be developed to improve long-term outcomes in severe ARDS survivors.

In conclusion, we reported that VV-ECMO treatment for severe ARDS does not seem to be associated with worse long-term cognitive and neuropsychological outcomes compared to patients not undergoing VV-ECMO. Nevertheless, the incidence of cognitive impairment and neuropsychological sequalae in severe ARDS patients remains high with consequences regarding quality of life and social health. Development of strategies for prevention and treatment is of major concern.

## Additional files

**Additional file 1: Table S1.** Evolution of SOFA score and respiratory parameters from day one (D1) to day 15 (D15).

**Additional file 2: Table S2.** Differences among the two groups according to the threshold used to define a cognitive impairment.

**Additional file 3: Table S3.** Indexes assessed by the WAIS-IV.

**Additional file 4: Table S4.** Health-related quality of life assessed by the SF-36 score.

**Additional file 5: Table S5.** Other information recorded during the interview with the ICU physician.

## Data Availability

The datasets used and/or analyzed during the current study are available from the corresponding author on reasonable request.

## Abbreviations

ICU: intensive care unit
ARDS: acute respiratory distress syndrome
SOFA: sepsis-related organ failure assessment score
VV-ECMO: venovenous extracorporeal membrane oxygenation
PTSD: post-traumatic stress disorder
P/F ratio: PaO_2_-to-FiO_2_ ratio
PEEP: positive end-expiratory pressure
WAIS-IV: Wechsler Adult Intelligence Scale, 4th edition
VCI: verbal comprehension index
PRI: perceptual reasoning index
WMI: working memory index
PSI: processing speed index
FSIQ: Full-Scale Intelligence Quotient
BDI-IA: Beck Depression Inventory
BAI: Beck Anxiety Inventory
IES: Impact of Event Scale
SF-36: Short-Form General Health Survey
IQR: interquartile range
SD: standard deviation
OR: odd ratios
CI: 95% confidence intervals
