# Supplementary material for: Long-term neurocognitive outcome is not worsened by of the use of venovenous ECMO in severe ARDS patients
Source: Ann Intensive Care. 2019 Jul 16;9:82. doi: 10.1186/s13613-019-0556-1 (PMC6635548; doi:10.1186/s13613-019-0556-1)
Supplement: Supplementary file 1 — Additional file 1: Table S1. Evolution of SOFA score and respiratory parameters from day one (D1) to day 15 (D15). [file 13613_2019_556_MOESM1_ESM.docx]

**Table S1:** Evolution of SOFA score and respiratory parameters from day one (D1) to day 15 (D15)

|  | **Non-ECMO**  **(n = 18)** | | | | | **ECMO**  **(n = 22)** | | | | |
| --- | --- | --- | --- | --- | --- | --- | --- | --- | --- | --- |
| **DAY** | **D1** | **D2** | **D3** | **D7** | **D15** | **D1** | **D2** | **D3** | **D7** | **D15** |
| **SOFA score** | 8 [6-12] | 8 [6-11] | 7 [6-11] | 7 [4-9] | 4 [3-5] | 8 [7-10] | 10 [8-11] | 10 [8-12] | 8 [7-11] | 5 [4-8] |
| **Lowest PaO2, mmHg** | 69 [62-79] | 73 [66-90] | 73 [62-84] | 69 [58-81] | 72 [66-81] | 73 [52-81] | 73 [69-88] | 68 [59-80] | 70 [64-84] | 73 [66-86] |
| **ACV** | 18 (100) | 18 (100) | 16 (89) | 8 (44) | 1 (6) | 17 (77) | 20 (91) | 17 (77) | 11 (50) | 6 (27) |
| **BiPAP/APRV** | 0 (0) | 0 (0) | 2 (11) | 5 (28) | 2 (22) | 5 (23) | 2 (9) | 5 (23) | 10 (45) | 9 (41) |
| **Pressure support** | 0 (0) | 0 (0) | 0 (0) | 5 (28) | 12 (67) | 0 (0) | 0 (0) | 0 (0) | 1 (5) | 5 (23) |
| **Spontaneous breathing** | 0 (0) | 0 (0) | 0 (0) | 0 (0) | 3 (17) | 0 (0) | 0 (0) | 0 (0) | 0 (0) | 2 (9) |
| **Highest FiO2, %** | 100 [70-100] | 63 [50-80] | 70 [50-100] | 50 [47.5-70] | 35 [30-43] | 100 [80-100] | 100 [35-100] | 80 [30-100] | 50 [40-75] | 50 [30-52.5] |
| **Tidal volume, ml/kg** | 6.4 [6-7.6] | 6.4 [5.9-7.7] | 5.9 [5.3-6.7] | 6.8 [6.4-7.9] | 7.5 [6.2-8.6] | 6.7 [5.5-7.5] | 3.0 [2.2-5.4] | 2.7 [2.1-5.4] | 5.7 [4.8-6.8] | 6.7 [5.6-7.5] |
| **No. patients** | 18 | 18 | 18 | 16 | 14 | 20 | 22 | 20 | 22 | 21 |
| **No. ECMO** | - | - | - | - | - | 3 | 14 | 17 | 16 | 10 |
| **Set PEEP, cmH2O** | 12 [10-15] | 14 [12-15] | 13 [10-16] | 12 [10-15] | 10 [8-12] | 12 [10-15] | 15 [10-18] | 16 [11-16] | 16 [12-18] | 12 [10-15] |
| **No. patients** | 18 | 18 | 18 | 17 | 15 | 21 | 22 | 19 | 22 | 21 |
| **No. ECMO** | - | - | - | - | - | 3 | 14 | 17 | 16 | 10 |
| **Plateau pressure, cmH2O** | 28 [27-30] | 28 [24-29] | 27 [24-30] | 28 [25-28] | *-* | 28 [26-30] | 26 [22-30] | 27 [22-32] | 27 [23-30] | 26 [25-30] |
| **No. patients** | 17 | 17 | 17 | 10 | 3 | 19 | 19 | 21 | 21 | 8 |
| **No. ECMO** | - | - | - | - | - | 3 | 14 | 17 | 16 | 10 |
| **Respiratory-system compliance, ml/cmH2O** | 28 [24-34] | 32 [25-40] | 25 [21-40] | 29 [25-33] | - | 26 [22-33] | 23 [11-25] | 23 [9-29] | 25 [18-31] | 26 [19-38] |
| **No. patients** | 17 | 17 | 17 | 10 | 3 | 19 | 19 | 19 | 21 | 8 |
| **No. ECMO** | - | - | - | - | *-* | 3 | 14 | 17 | 16 | 10 |

Data are provided as numbers (%) for categorical variables and as medians [25^th^-75^th^ percentiles] for continuous variable

*SOFA,* sequential organ failure assessment, *ACV*, assist control ventilation, *BiPAP* biphasic intermittent positive airway pressure, *APRV*, airway pressure release ventilation
