# Supplementary material for: Long-term neurocognitive outcome is not worsened by of the use of venovenous ECMO in severe ARDS patients
Source: Ann Intensive Care. 2019 Jul 16;9:82. doi: 10.1186/s13613-019-0556-1 (PMC6635548; doi:10.1186/s13613-019-0556-1)
Supplement: Supplementary file 2 — Additional file 2: Table S2. Differences among the two groups according to the threshold used to define a cognitive impairment. [file 13613_2019_556_MOESM2_ESM.docx]

## **Table S2**: Differences among the two groups according to the threshold used to define a cognitive impairment

| **Variable** | **Non-ECMO**  **(n = 18)** | **ECMO**  **(n = 22)** | **P-value** |
| --- | --- | --- | --- |
| ≥1 index > 1 SD | 10 (56) | 12 (55) | 0.95 |
| ≥2 indexes > 1 SD | 5 (28) | 7 (32) | 0.78 |
| ≥2 indexes > 1SD or ≥1 index > 1.5 SD | 6 (33) | 7 (32) | 0.92 |

Data are provided as numbers (%)

*SD*, standard deviation
