# Supplementary material for: Long-term neurocognitive outcome is not worsened by of the use of venovenous ECMO in severe ARDS patients
Source: Ann Intensive Care. 2019 Jul 16;9:82. doi: 10.1186/s13613-019-0556-1 (PMC6635548; doi:10.1186/s13613-019-0556-1)
Supplement: Supplementary file 3 — Additional file 3: Table S3. Indexes assessed by the WAIS-IV. [file 13613_2019_556_MOESM3_ESM.docx]

## **Table S3:** Indexes assessed by the WAIS-IV

| **Variable** | **Non-ECMO**  **(n = 18)** | **ECMO**  **(n = 22)** | **P-value** |
| --- | --- | --- | --- |
| Verbal Comprehension Index | 88 [81-102] | 95 [87-102] | 0.34 |
| Perceptual Reasoning Index | 95 [86-102] | 93 [87-103] | 0.93 |
| Working Memory Index | 96 [87-106] | 89 [83-100] | 0.25 |
| Processing Speed Index | 96 [85-102] | 93 [83-98] | 0.56 |
| Full Scale Intelligence Index | 94 [81-97] | 91 [83-100] | 0.99 |

Data are provided as medians [25^th^-75^th^ percentiles]
