# Supplementary material for: Long-term neurocognitive outcome is not worsened by of the use of venovenous ECMO in severe ARDS patients
Source: Ann Intensive Care. 2019 Jul 16;9:82. doi: 10.1186/s13613-019-0556-1 (PMC6635548; doi:10.1186/s13613-019-0556-1)
Supplement: Supplementary file 4 — Additional file 4: Table S4. Health-related quality of life assessed by the SF-36 score. [file 13613_2019_556_MOESM4_ESM.docx]

## **Table S4:** Health-related quality of life assessed by the SF-36 score

| **Variables** | **Non-ECMO**  **(n = 18)** | **ECMO (n = 22)** | **P-value** |
| --- | --- | --- | --- |
| Physical functioning | 60 [25-78] | 65 [28-88] | 0.66 |
| Social functioning | 50 [31-88] | 63 [44-88] | 0.60 |
| Physical role | 25 [0-75] | 25 [0-100] | 0.64 |
| Emotional role | 33 [0-100] | 67 [0-100] | 0.71 |
| Mental health | 64 [40-82] | 62 [46-84] | 0.75 |
| Vitality | 45 [25-50] | 50 [36-65] | 0.21 |
| Pain | 72 [22-90] | 62 [41-84] | 0.91 |
| General health | 50 [28-65] | 47 [29-65] | 0.93 |
| Physical-component score | 39 [28-49] | 36 [32-53] | 0.58 |
| Mental-component score | 44 [29-50] | 45 [32-55] | 0.62 |

Data are provided as medians [25^th^-75^th^ percentiles]

*The domains of the Medical Outcomes Study 36-item Short-Form General Health Survey (SF-36) are defined as follows: physical functioning, the extent to which health limits physical activity; physical role, the extent to which physical health interferes with work or limits activity; pain, the intensity of pain and the effect of pain on patient’s ability to work; general health, patient’s own evaluation of his or her health or health outlook; vitality, the degree of energy the patient has; social functioning, the extent to which health or emotional problems interfere with social activities; emotional role, the extent to which emotional problems interfere with work or activities; and mental health, general mental health. Scores for each domain can range from 0 to 100; higher scores denote a better health-related quality of life.*
