# Supplementary material for: Long-term neurocognitive outcome is not worsened by of the use of venovenous ECMO in severe ARDS patients
Source: Ann Intensive Care. 2019 Jul 16;9:82. doi: 10.1186/s13613-019-0556-1 (PMC6635548; doi:10.1186/s13613-019-0556-1)
Supplement: Supplementary file 5 — Additional file 5: Table S5. Other information recorded during the interview with the ICU physician. [file 13613_2019_556_MOESM5_ESM.docx]

## **Table S5:** Other information recorded during the interview with the ICU physician

| **Variable** | **Non-ECMO**  **(n = 18)** | **ECMO**  **(n = 22)** | **P-value** |
| --- | --- | --- | --- |
| **Site of visit, n (%)** |  |  | 0.054 |
| Hospital | 17 (94) | 15 (68) |  |
| Home | 1 (6) | 7 (32) |  |
| Rehospitalization since discharge from hospital, n (%) | 10 (56) | 10 (46) | 0.53 |
| ***Need assistance at home, n (%)*** | 10 (56) | 14 (64) | 0.60 |
| < 3 months | *6 (55)* | *5 (39)* |  |
| 3-9 months | 2 (18) | 5 (39) |  |
| Ongoing | 3 (27) | 3 (23) |  |
| Marital status unchanged, n (%) | 15 (88) | 15 (68) | 0.14 |
| Actual BMI, kg/m2 | 27 [21-30] | 26 [22-30] | 0.63 |
| Change in weight, kg | -6,5 [-12-1] | 0 [-15-5] | 0.56 |
| Still smoking, n (%) | 3 (17) | 6 (27) | 0.48 |
| Alcohol, n (%) | 6 (33) | 7 (32) | 0.92 |
| Mood changes, n (%) | 10 (56) | 10 (48) | 0.62 |
| Sleep disorders, n (%) | 9 (56) | 12 (71) | 0.39 |
| Dyspnea, n (%) | 4 (22) | 6 (27) | 0.99 |
| Ongoing psychological follow-up, n (%) | 2 (11) | 0 (0) | 0.20 |
| Psychotropic drug treatment, n (%) | 6 (33) | 4 (18) | 0.30 |
| Medical follow-up recommendation by the ICU physician, n (%) | 4 (22) | 7 (32) | 0.50 |
| Psychological follow-up recommendation by the ICU physician, n (%) | 9 (50) | 11 (50) | 0.99 |

Data are provided as numbers (%) for categorical variables and as medians [25^th^-75^th^ percentiles] for continuous variables.
